# Godey’s Lady’s Book

**Published:** 1867-02

**Authors:** 


Godey's Lady's Book.—This popular and attractive magazine is placed regularly upon our table. A physician can, perhaps, do very well without some of the current popular literature of the day, but no well regulated family should be without the various magazines of this character, especially Godey's Lady's Book.

---

Several valuable books have been received, but the due acknowledgment has been unavoidably crowded out; they will be duly noticed in our next number.

